# *Lactobacillus rhamnosus* HN001 Attenuates Allergy Development in a Pig Model

**DOI:** 10.1371/journal.pone.0016577

**Published:** 2011-02-28

**Authors:** Debra J. Thomas, Robert J. Husmann, Mauricio Villamar, Timothy R. Winship, Rachael H. Buck, Federico A. Zuckermann

**Affiliations:** 1 Abbott Nutrition, Columbus, Ohio, United States of America; 2 Department of Pathobiology, College of Veterinary Medicine, University of Illinois, Urbana, Illinois, United States of America; Centre de Recherche Public de la Santé (CRP-Santé), Luxembourg

## Abstract

**Background:**

Probiotics have been studied as immunomodulatory agents of allergy. Several human probiotic trials tracking the development of eczema and other forms of allergy have yielded inconsistent results. A recent infant study demonstrated that pre and postnatal *Lactobacillus rhamnosus* HN001 (HN001) supplementation decreased the prevalence of eczema and IgE associated eczema. However, the influence of HN001 on the incidence of wheeze, asthma, and/or other allergic manifestations has yet to be reported.

**Objective:**

This study was conducted to determine the effects of the probiotic HN001 on the development of allergic lung disease in a pig model.

**Methods:**

Allergy was induced by a series of subcutaneous and intratracheal sensitizations with *Ascaris suum* allergen (ASA) during a six week time frame in post-weanling pigs supplemented daily with HN001, or without supplementation. One week following final sensitization intradermal skin tests and respiratory challenges were conducted.

**Results:**

In response to intradermal and respiratory challenges, ASA-sensitized pigs fed HN001 had less severe skin flare reactions, smaller increases in pleural pressure, and trends towards lower changes in arterial oxygen and carbon dioxide partial pressure levels compared to control pigs. The frequency of ASA-specific IFN-γ-secreting peripheral blood mononuclear cells, as well as the amount of IL-10 produced by ASA-specific cells, was of greater magnitude in probiotic-fed pigs compared to control animals. These observations suggest that differences in clinical responses to the allergen challenges may be related to probiotic-induced modulation of Th1 (IFN-γ) and regulatory (IL-10) cytokine expression.

**Conclusions:**

Probiotic supplementation decreased the severity of allergic skin and lung responses in allergen-sensitized pigs with a corresponding increase in IFN-γ expression. A similar correlation between certain allergic responses and increased IFN-γ expression has been reported in human clinical studies of allergy; this pig model of allergy may be indicative of potential probiotic modulation of allergic lung disease in humans.

## Introduction

Probiotics have been studied as mucosal immune modulators targeting allergy outcomes. Several human probiotic trials tracking eczema and other forms of allergy have yielded inconsistent results [1], [2], [3], [4], [5], [6]. Interestingly, two studies using the same probiotic, *Lactobacillus rhamnosus* GG, with similar study designs, resulted in different outcomes; either a decrease in eczema was observed [1] or no probiotic benefit was reported [5]. Furthermore, a follow-up report indicated the initial decrease in eczema in at risk infants supplemented with the probiotic *Lactobacillus rhamnosus* GG was accompanied by the tendency of increased wheeze in this group at 7 years of age [7]. In a study using *Lactobacillus acidophilus* LAVRI-A1 there was no probiotic-associated decrease in atopic dermatitis, but there was significantly more allergen sensitization and an increase in wheeze in supplemented infants [4]. In a more recent infant clinical study, *Lactobacillus rhamnosus* HN001 (HN001) was supplemented prenatally to expectant mothers of at risk infants, and antenatally to their infants. Mothers received the probiotic supplementation from 2-4 weeks prior to delivery and continued the supplementation through the next 6 months if breast-feeding. Regardless, infants were supplemented from birth through the first 2 years of life. Results from this trial indicated that HN001 decreased the prevalence of eczema and of IgE-associated eczema in the study population at 24 months of age compared to age-matched controls (*p* = 0.01 and *p* =  0.04 respectively) [8].

The influence of HN001 on the incidence of wheeze, asthma, and/or other allergic manifestations has yet to be reported. Therefore, our objective was to determine if this probiotic (HN001) would modulate allergic reactions in a positive or negative manner. To this end, we modified a porcine model used to study allergic airway reactions [9] so that the effect of HN001 on the immediate lung and skin allergic responses could be evaluated.

Briefly, starting at 3 weeks of age, pigs were divided into two groups, one of which received supplementation with the probiotic HN001. At this time, both groups of animals were injected subcutaneously with allergen from the endoparasitic roundworm, *Ascaris suum*. During the ensuing 6 weeks, all animals were sensitized three more times via subcutaneous and twice by intratracheal routes with the allergen. At seven weeks from the initial sensitization, the pigs were challenged via intradermal and intratracheal administration of the allergen. Using this pig model of allergy we demonstrated the ability to induce allergic manifestations of immediate skin hypersensitivity and allergic lung disease in pigs, as well as a reduction in the severity of both of these allergic manifestations via probiotic intervention.

## Materials and Methods

### Ethics Statement

This study was approved by the Institutional Animal Care and Usage Committee of the University of Illinois, number 09312, and conducted in compliance with local and federal guidelines regulating laboratory animal care and housing.

### Animals

Twelve specific pathogen free hybrid (Duroc [boar] x Yorkshire/Landrace [sow]) pigs of comparable weights were obtained from six litters at the University of Illinois Veterinary Medicine Research Farm. Pigs were sow-reared for the first 21 days of life, then weaned to creep feed, and from day 35 forward fed adult pig chow (University of Illinois Feed Mill, Champaign, IL). Pigs were housed in accordance with standard animal usage, maintained on 12 hour light/dark cycles, and had free access to water and study feeds. Pigs were weighed after weaning and at various times during the ensuing 49 days as an assessment of health status.

### Probiotic and probiotic treatment

At the onset of weaning (study day 21), pigs were assigned to either the control (n = 6) or probiotic-treated group (n = 6) with equal litter representation between study groups. Members of the latter group were individually fed a daily dose of ∼1×10^10^ colony forming units of the probiotic, HN001 (Danisco, Madison, WI) that had been mixed with a small amount of vanilla pudding. Because pigs like vanilla flavoring, they immediately ate the probiotic-modified pudding assuring that each pig received its daily probiotic dose. Control group animals received vanilla pudding lacking the probiotic.

### Allergy induction

This model of allergic lung disease is a modification of that reported by Fornhem *et al.* [9]. Pigs were sensitized on study days 21, 35, and 49 by subcutaneous injection into the posterior section of the hind leg with 1.0 mg *Ascaris suum* allergen (ASA, Greer Laboratories, Lenoir, NC) plus alum (0.5 mL of 10% alum per 10 mg of protein) in 2 mL of saline. Moreover, on study days 49 and 63, 1.0 mg ASA without alum in 5 mL saline was administered to each animal, instilled at the level of their lower trachea, with a nebulizer, via an endotracheal tube. These procedures were performed under anesthesia induced by an intramuscular injection of a solution containing Telazol®, ketamine, and xylazine at 4.4, 2.2, and 2.2 mg/kg body weight, respectively.

### Intradermal ASA challenge and evaluation of the response

Following an overnight fast, pigs were anesthetized on study days 45 and 70 as described above. Challenge consisted of a line of intradermal injections at 11 sites from anterior to posterior on pigs' abdomens. One hundred microliters of each 2-fold ASA serial dilution, ranging from 400 µg/mL to 0.75 µg/mL, as well as with saline alone, was injected per site. After 20 minutes, reactions to the ASA were measured by using ultra-test calipers (General MG, Japan) to determine the diameter of the flare at the site of allergen or saline injection.

### Respiratory ASA challenge and evaluation of the response

Respiratory function assessments were performed on study day 70. Anesthesia was induced as previously described and maintained by a continuous intravenous infusion of a mixture of xylazine (1 mg/mL), ketamine (1 mg/mL) and guaifenesin (5% v/v) dripping at an approximate rate of 1 mL/kg/h. One of the control animals had an unexpected and unexplained reaction to the anesthetic; further testing was not conducted on this pig. Anesthesia was initiated approximately 1 hour prior to respiratory challenge to enable arterial and venous vessel catheterization (saphenous artery and ear vein, respectively) tracheal intubation, esophageal balloon placement, and measurement of the intradermal responses to ASA. Balloon placement consisted of a balloon being inserted into the pig's esophagus through the animal's mouth and positioned mid-thorax. After being filled with 1–2 cc of air, its correct placement was verified by obtaining a representative pleural pressure curve, according to standard technique [10].

Respiratory challenges were performed within 10 minutes after recording intradermal allergic responses. Challenges were conducted by using a portable ultrasonic nebulizer. Two point five milligrams of ASA in 0.5 mL of saline was administered to the lower airways of spontaneously breathing anesthetized pigs via an endotracheal tube during a 1 minute period. Blood samples taken from a catheter placed in the saphenous artery were collected at 0, 2, 5, 10, 20, and 30 minutes post-respiratory challenge. Levels of arterial blood gases (pO_2_ and pCO_2_) in these samples were determined by using a blood gas analyzer (Rapidlab 855, Bayer [now Siemens Medical Solutions Diagnostics, Tarrytown, NY]). As a precaution, injectable epinephrine was available to counteract anaphylactic reactions, however; the use of epinephrine was not necessary.

Lung function was assessed as previously described [11] with modifications. Briefly, tracheal airflow was measured with a pneumotachygraph (TSD137G, Biopac Systems, Inc. Goleta, CA), connected to a differential pressure transducer (TSD160B, Biopac Systems). Pleural pressure (Pp) was measured via the esophageal balloon connected to a pressure transducer (TSD104A, Biopac Systems). The airflow and Pp signals were amplified using a transducer amplifier (DA100C, Biopac Systems) and then sent to an acquisition and analysis system (MP150, Biopac Systems) which recorded and analyzed the signals continuously from 2 minutes before to 30 minutes after the respiratory challenge. These recordings were used to calculate the lung resistance (RL; mmHg/mL second) and dynamic lung compliance (Cdyn; mL/mmHg) using the AcqKnowledge software (Biopac Systems, Inc). Average values from data encompassing at least 30 seconds of continuous recording were calculated at 0, 2, 5, 10, 20 and 30 minutes after challenge.

### Quantitation of cytokine secretion by peripheral blood mononuclear cells (PBMCs) by ELISA

Blood was collected from the pigs' jugular veins on study days 35, 49, 63, and 70 and PBMCs from these samples were isolated using standard density gradient procedures as described previously [12]. Ten million cells from each animal were cultured in individual wells of a 24-well plate in RPMI medium supplemented with 5% fetal porcine serum [12] in the presence or absence of 10 µg/mL ASA for 24 hours at 37°C in a 5% CO_2_ atmosphere. Afterwards, the culture supernatants were collected and stored at −20°C until tested for the presence of interleukin (IL)-4 and IL-10 by using specific ELISA CytoSet components from Invitrogen (Carlsbad, CA.) according to manufacturer's instructions.

### Detection of interferon (IFN)-γ secreting cells in PBMCs by ELISPOT

The frequencies of IFN-γ secreting PBMCs (PBMCs isolated as described above) on study days 35, 49, 63, and 70 were determined by adapting a previously described ELISPOT assay [12] for use with ASA stimulation. This method utilizes unlabeled monoclonal antibody (mAb) P2G10 and biotin-labeled, mAb P2C11 (BD/Pharmingen, San Diego, CA) that are specific for different epitopes of porcine IFN-γ [13]. Briefly, the bottoms of 96-well Immulon II plates (Dynatech, Inc.) were coated with 50 µL per well of 10 µg/mL mAb P2G10 in 0.1 M carbonate buffer, pH 9.6. After a 4°C overnight incubation, the wells were washed three times with sterile PBS and then incubated with 50 µL of RPMI medium supplemented with 5% fetal porcine serum for 2 hours at 37°C in a 5% CO_2_ atmosphere. Fifty thousand PBMCs were added per well and exposed to 10 µg/mL ASA at 37°C in a 5% CO_2_ atmosphere for 20 hours. Cells were then removed by washing the wells six times with PBS containing 0.05% Tween-20. Fifty microliters of 0.5 µg/mL biotin-labeled mAb P2C11 in 0.05% PBS–Tween was added to each well and plates were incubated for 1 hour at 37°C. After again washing six times, 50 µL of 0.31 µg/mL streptavidin–horseradish peroxidase (Zymed; SanFrancisco, CA) was added to each well. Plates were incubated for 1 hour at 37°C. Excess streptavidin–horseradish peroxidase was removed by washing the wells three times; 50 µL of 3,3′,5,5′-Tetramethylbenzidine membrane peroxidase substrate (Kirkegaard & Perry Laboratories, Gaithersburg, MD) was added to each well for 30 minutes at 37°C. Hydrolysis of this compound results in the development of blue spots whose size and intensity are directly proportional to the amount of bound IFN-γ [12]. The frequency of ASA-specific IFN-γ-producing cells in each sample was determined by averaging the number of blue spots in duplicate wells.

### Lung assessments

**Figure 1:**
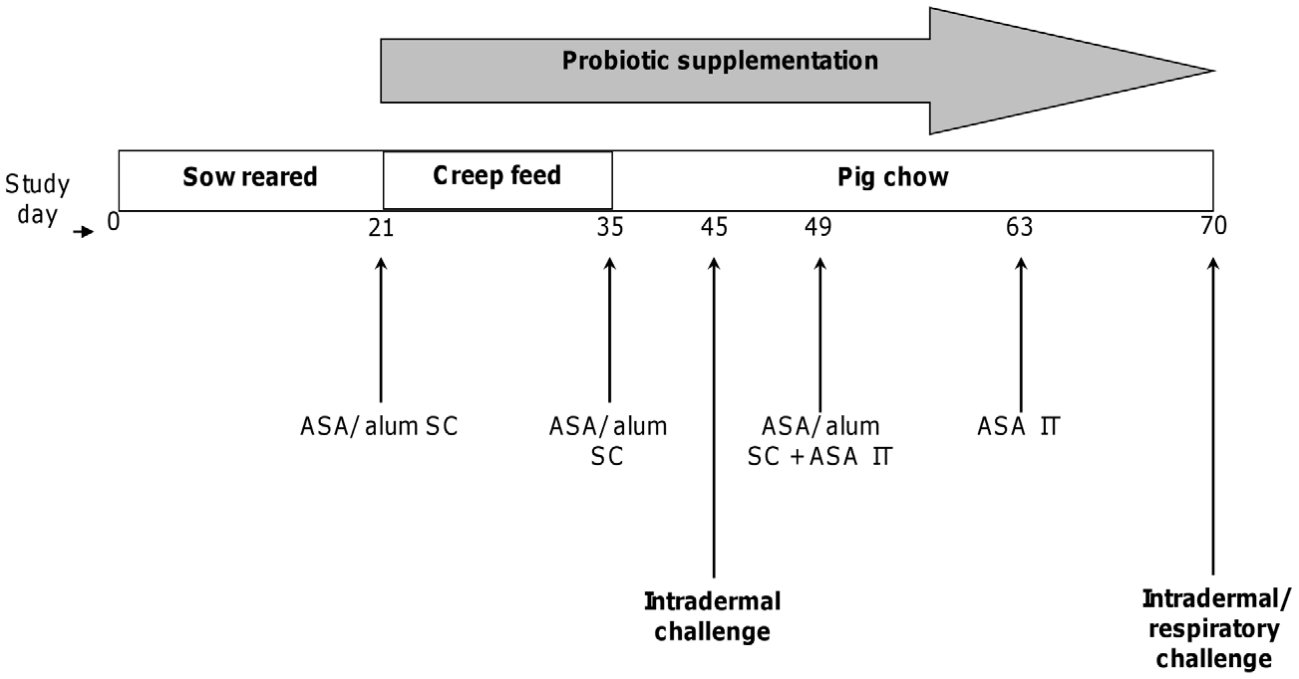
Schematic representation of study design. The entire 70 day process involving the transtition from weaning to adult food intake, the onset and continuation of probiotic supplementation, the subcutaneous (SC) and intratracheal (IT) introduction of ASA plus alum and ASA without alum (ASA/alum and ASA, respectively) at 1 mg doses/pig, and the intradermal and respiratory challenges with ASA is delineated.

Twenty-four hours after respiratory challenge, pigs were anesthetized by intramuscular injection, as described above, and then euthanized by intravenous injection of a 30 mL bolus of a saturated solution of potassium chloride. Necropsy immediately followed. Lungs (without trachea and lymph nodes) were removed, weighed, and the accessory lobe was lavaged with approximately 120 mL of sterile phosphate buffered saline. Cell counts from bronchoalveolar lavage (BAL) fluid were performed with a Cell-Dyn 3700 (Abbott Diagnostics, Chicago, IL) and leukocyte differential counts were determined by microscopic analyses of Giemsa stained cytospin preparations. See Figure 1 for study design.

### Statistical Analysis

Repeated measures analysis of variance of intradermal response data between groups was performed using SAS software, Version 9.1 (Cary, NC). Student's t tests for each allergen concentration were also performed between groups (unpaired, 2-tailed, equal variance, Excel Software 2003). Student's t tests were performed within groups comparing the extent of skin flare reactions at study day 45 to study day 70 (paired, 2-tailed, equal variance, Excel Software 2003).

Lung function was assessed to determine recovery from challenge and/or severity of reaction. Each measurement at 2, 5, 10, 20, and 30 minutes post-respiratory challenge was compared to the respective baseline result (0 minutes post-challenge; each pig served as its own control) to identify change over time within each group (Student's t tests, paired, 2-tailed, equal variance). As the biological variation in allergic phenotype among litters is typically great, it was necessary to normalize the biological variation of the lung assessments for comparisons between the two groups. Therefore, the percent change of each lung function assessment was analyzed by repeated measures analysis of variance and at each time-point by Student's t test (unpaired, 2-tailed, equal variance). The percent change was calculated by subtracting the baseline value from the value at a given time-point, dividing by the baseline value, and then multiplying by 100%.

Cytokine data were analyzed by Student's t test (paired, 2-tailed, equal variance) comparing days 49, 65, and 70, to day 35 within groups and also between groups by repeated measures of variance analysis and by Student's t tests (unpaired, 2-tailed, equal variance) at each time-point. Due to the small number of animals per group, statistically significant values (*p*-value ≤0.05) or trends/tendencies towards significance (0.051≤*p*-value ≤0.1) were interpreted as suggestive.

## Results

### Characterization of skin and lung histological changes in non-sensitized and ASA-sensitized pigs after exposure to ASA

**Figure 2:**
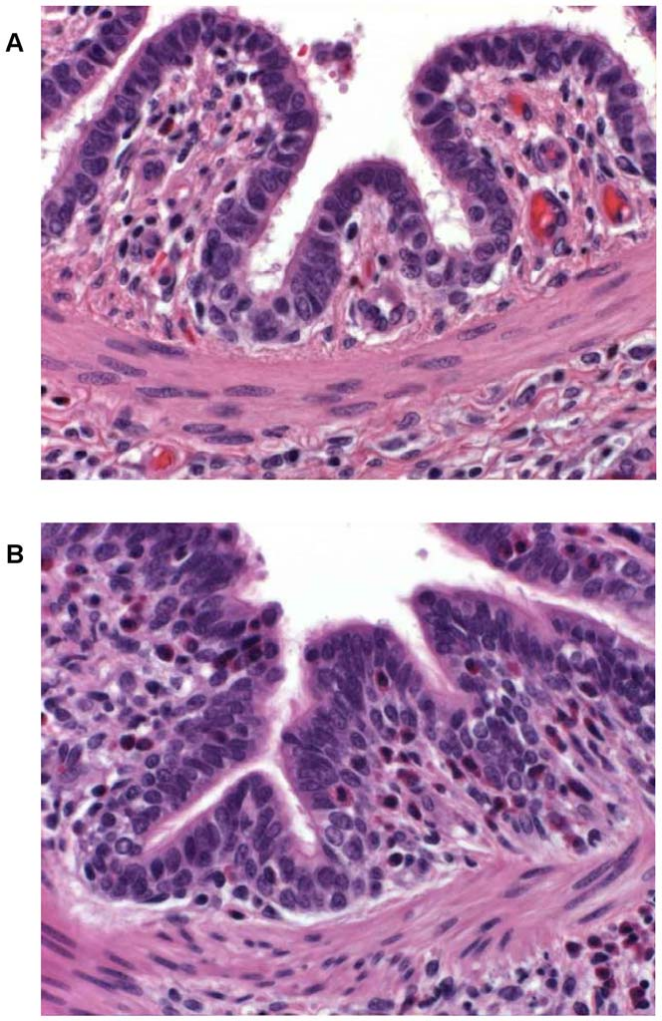
Lung tissue 24 hours post-respiratory challenge with ASA. Lungs were removed from a non-sensitized (A) and an ASA-sensitized (B) pig 24 hours after respiratory challenge, fixed in formalin, stained with hematoxylin and eosin, and examined with a light microscope (400X magnification). An increased cellular infiltrate, composed mainly of eosinophils (cells with a pink cytoplasm surrounding a blue nucleus), in airway submucosa and lamina propria is apparent in the tissue from the ASA-sensitized animal as compared to that from the naïve pig.

In previous non-intervention studies pigs sensitized with ASA and challenged intradermally or intratracheally with ovalbumin, or animals mock sensitized with phosphate buffered saline and challenged in a similar manner with ASA, did not exhibit dermal or respiratory reactions (data not shown). In contrast, an ASA intradermal challenge to ASA-sensitized animals is associated with a typical dermal inflammatory response that is characterized by reddening of the injection site within 5 minutes of exposure. The flare intensity increases, peaking by 20 minutes, and gradually wanes thereafter. To establish the nature of the skin flare response, tissue samples from reactive sites of several ASA-sensitized pigs challenged intradermally with 40 µg ASA 20 minutes earlier were stained with hematoxylin and eosin and examined microscopically. Histological evaluation of reactive skin sites indicated marked dermal and subcutaneous edema as well as perivascular infiltration by lymphocytes and eosinophils (data not shown). A similar examination of lung tissue samples from ASA intratracheally challenged pigs demonstrated the infiltration of eosinophils into the lamina propria of airway epithelium in ASA-sensitized animals only and not in ASA-naïve pigs (Figure 2A & B). Eosinophil infiltration of tissues is a hallmark of allergic responses and lung eosinophilia has been implicated as a factor of lung remodeling in asthma [14], [15].

### Probiotic supplementation diminished allergic skin flare responses to intradermal challenge with ASA in ASA-sensitized pigs

**Figure 3:**
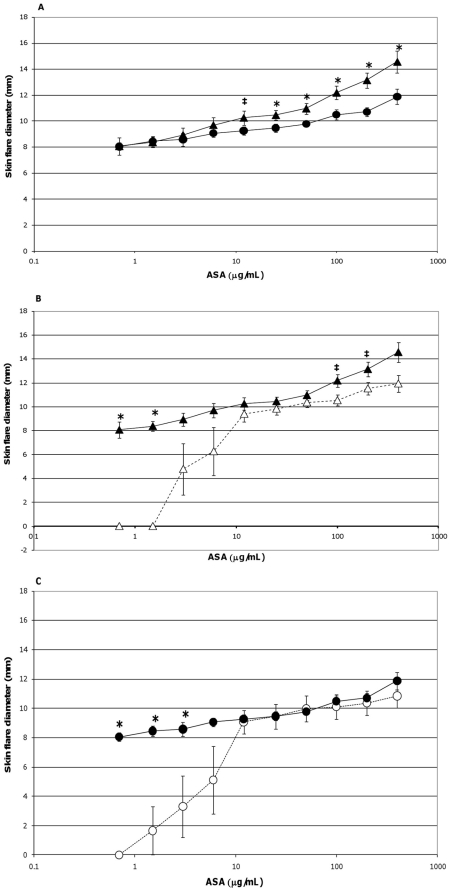
Skin flare reactions 20 minutes post-intradermal challenge with ASA. ASA-sensitized pigs were intradermally inoculated with the indicated amounts of ASA. After 20 minutes the diameters of the resultant skin flares at the sites of inoculation were measured. Results are expressed as mean ± standard error of mean (SEM) for the respective group of pigs (n = 6). (A) Measurements made on study day 70 for control (triangles) and probiotic-fed (circles) groups. Differences between the responses of the two groups are indicated by * (*p*≤0.05) or ‡ (0.051≤*p*≤0.1) and are not corrected for multiple comparison (Student's t test, unpaired). (B) Measurements made on study days 45 (open triangles) and 70 (filled triangles) for the control group. Differences between the responses of this group on day 45 versus day 70 are indicated by * (*p*≤0.05) or ‡ (0.051≤*p*≤0.1) (Student's t test, paired). (C) Measurements made on study days 45 (open circles) and 70 (filled circles) for the probiotic-fed group. Differences between the responses of this group on day 45 versus day 70 are indicated by * (*p*≤0.05) (Student's t test, paired).

In the current study, the extent of skin flare responses in the ASA-sensitized pigs to intradermal challenges with ASA were compared on a group and temporal basis to determine any influence due to probiotic supplementation or additional exposure to the allergen. There was no detectable difference in the degree of skin flare responses between the two groups on study day 45. However, after an interval of 25 days (study day 70) which encompassed two additional exposures to ASA (Figure 1), control pigs developed more severe reactions to allergen doses of 400 µg/mL (*p* = 0.024), 200 µg/mL (*p* = 0.005), 100 µg/mL (*p* = 0.027), 50 µg/mL (*p* = 0.024), and 25 µg/mL (*p = 0.066* [trend]) than probiotic fed animals (Figure 3A); when adjusting for multiple comparisons, the *p*-value of the 200 µg/mL dose retained significance. Responses between the two groups to lower allergen doses were not significantly different (Figure 3A).

The additional exposure to ASA also impacted the severity of skin flare responses of control pigs; a trend for increased skin flare responses was seen at allergen doses of 200 µg/mL (*p* = 0.059) and 100 µg/mL (*p* = 0.068) when measured on study day 70 as compared to study day 45 (Figure 3B). In contrast, at these concentrations the intensity of this response in probiotic-fed pigs remained relatively consistent between study days 45 and 70 (Figure 3C). Thus, probiotic supplementation appeared to attenuate a heightening in severity of skin flare response to the largest amounts of allergen (Figure 3C). For both groups there was an increase in skin flare size at the latter study day to allergen doses of 1.5 and 0.75 µg/mL (*p* = <0.001,in both groups at both concentrations, Figures 3B and 3C). Moreover, for the probiotic group only, an enhancement was also noted at the 3 µg/mL concentration (*p* = 0.032, Figure 3C). This augmentation in the allergic response in relation to the low end of both dose response curves indicated the sensitivity of the skin flare responses increased with repeated ASA exposure for both groups and that probiotic supplementation did not influence sensitivity to the allergen.

### Probiotic supplementation diminished allergic lung responses to intratracheal challenge with ASA in ASA-sensitized pigs

Changes in lung function resulting from the allergen challenge were assessed by calculating lung resistance (RL) and dynamic lung compliance (Cdyn) using Pp and air flow recorded values. To determine the level of peripheral blood oxygenation, arterial blood gas (pO_2_ and pCO_2_) levels were also measured. Simplistically, lung resistance is a measure of the ease in which air flows through the respiratory structures, and lung compliance is the ability of the lung to expand or the “flexibility” of the lung. Increased lung resistance and decreased lung compliance indicate the lung has lost “flexibility” or has become “stiffer” due to fibrous tissue, and in the case of an asthmatic episode, cellular infiltrates, edema, and bronchi constriction. Increased Pp indicates labored breathing or respiratory distress, while a drop in arterial pO_2_ accompanied by an increase in arterial pCO_2_ indicates the lung has diminished capacity to oxygenate and remove CO_2_ from the blood.

Acute and adverse changes in the lung function of pigs after intratracheal ASA challenge were observed for both groups although differences in lung function recovery between the two groups were noted. A marked increase in RL was observed in all animals within 5 minutes after being challenged with ASA. While these values for the probiotic-fed group were not different from baseline by 20 minutes post-challenge, the RL for control animals remained elevated through the 30 minute monitoring period (Table 1). As with RL, Pp, pO_2_ (trend for difference at 30 minute time-point), and pCO_2_ levels returned to baseline values sooner for the probiotic-fed group than the control group (Table 1). Thus, probiotic supplementation was associated with a faster return to normal lung function.

**Table 1:** Lung function recovery: assessments post-respiratory challenge compared to baseline.

Lung function assessment	Study Group	2 minutes post-challenge (*p*-value)	5 minutes post-challenge (*p*-value)	10 minutes post-challenge (*p*-value)	20 minutes post-challenge (*p*-value)	30 minutes post-challenge (*p*-value)
Lung resistance	Control (n = 5)	*0.070*	**0.040**	**0.004**	**0.026**	**0.035**
	Probiotic (n = 6)	**0.027**	*0.056*	*0.058*	ND	ND
Lung compliance	Control (n = 5)	ND	**0.024**	**0.032**	ND	ND
	Probiotic (n = 6)	**0.039**	**0.035**	**0.047**	*0.058*	ND
Pleural pressure	Control (n = 5)	*0.070*	**0.005**	**0.015**	**0.032**	**0.006**
	Probiotic (n = 6)	**0.026**	**0.037**	*0.069*	ND	ND
pO_2_	Control (n = 5)	**0.026**	**0.001**	**<0.001**	**0.005**	**0.018**
	Probiotic (n = 6)	**0.010**	**0.002**	**<0.001**	**0.005**	*0.065*
pCO_2_	Control (n = 5)	**0.052**	*0.057*	**0.031**	ND	ND
	Probiotic (n = 6)	ND	ND	ND	ND	ND

Bolded values  =  significant differences from baseline, italicized values  =  trends for differences from baseline, ND  =  not different (significant or trend) from baseline indicating recovery of lung function.

**Figure 4:**
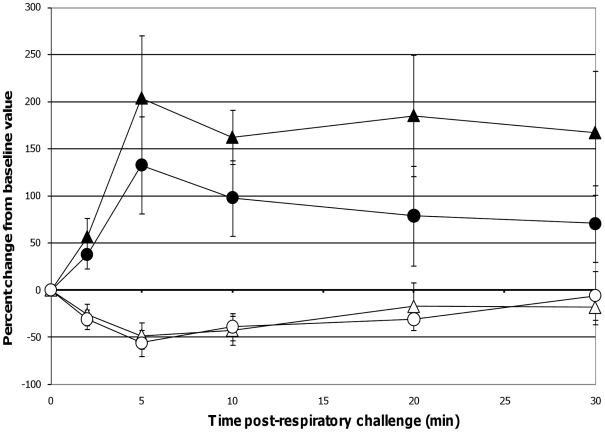
Changes in RL and Cdyn post-respiratory challenge with ASA. Lung function parameters of ASA-sensitized pigs were continuously monitored from 2 minutes before to 30 minutes after animals were intratracheally challenged with ASA on study day 70. Based on at least a 30 second interval of these recordings around the indicated times post-respiratory challenge, the average values for RL and Cdyn were determined and are presented as the mean ± SEM percent change relative to the baseline (0 minutes) value for the probiotic-fed pigs (n = 6) and control pigs (n = 5). Average percent changes for RL and Cdyn in probiotic-fed pigs (filled and open circles, respectively) and in control animals (filled and open triangles, respectively) are shown.

**Figure 5:**
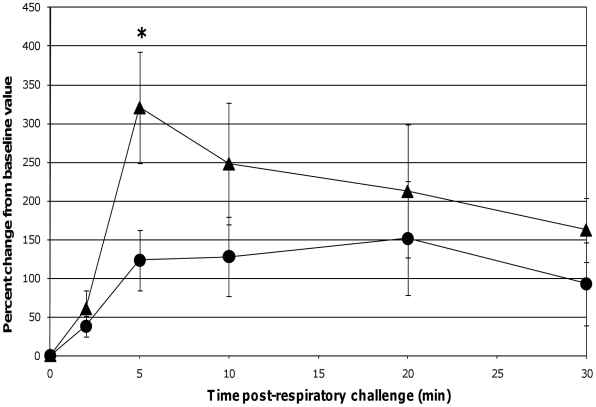
Changes in Pp post-respiratory challenge with ASA. The Pp of ASA-sensitized pigs was continuously monitored from 2 minutes before to 30 minutes after animals were intratracheally challenged with ASA on study day 70. Based on at least a 30 second interval of these recordings around the indicated times post-respiratory challenge, the average values for Pp were determined and are presented as the mean ± SEM percent change relative to the baseline (0 minutes) value for the probiotic-fed pigs (n = 6) and control pigs (n = 5). Average percent changes for Pp in probiotic-fed pigs (filled circles) and in control animals (filled triangles) are shown. A significant difference between the relative percent change in Pp for the probiotic-fed and control groups is indicated by * (*p* ≤0.05) (Student's t test, unpaired).

**Figure 6:**
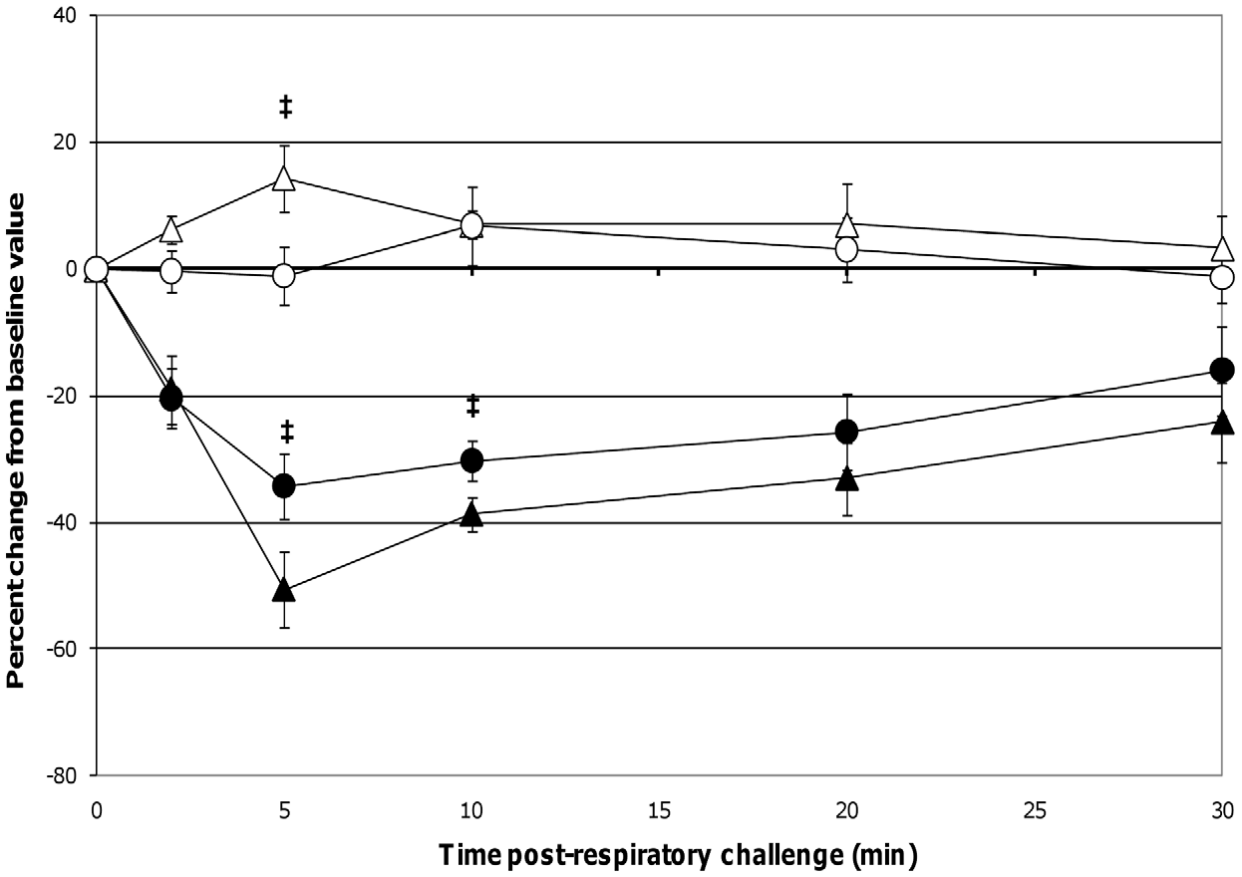
Changes in pO_2_ and pCO_2_ levels post-respiratory challenge with ASA. Saphenous arterial blood samples were collected from ASA-sensitized pigs at the indicated times prior to and after intratracheal ASA challenge on study day 70. Blood gas levels (pO_2_ and pCO_2_) from these samples were determined and the average values are presented as the mean ± SEM percent change relative to the baseline (0 minutes) value for the probiotic-fed pigs (n = 6) and control pigs (n = 5). Average percent changes for pO_2_ levels in probiotic-fed pigs (filled circles) and in control animals (filled triangles) and for pCO_2_ levels in probiotic-fed pigs (open circles) and in control animals (open triangles) are shown. Differences between the relative percent change in pO_2_ and pCO_2_ levels for the probiotic-fed and control groups is indicated by ‡ (0.051≤*p* ≤0.1) (Student's t test, unpaired).

When comparing lung function assessments between groups, the probiotic-fed pigs exhibited a lower average percent change in RL from baseline at all time-points as compared to the control group, but due to the variability among animals, especially in the control group, the group means were not statistically significant (Figure 4). No differences in lung Cdyn were noted between groups (Figure 4). Five minutes post-respiratory challenge the percent change in Pp (*p* = 0.032, Figure 5), pO_2_ levels (*p* = 0.075, Figure 5) and pCO_2_ levels (*p* = 0.058, Figure 6) was less or tended to be less in the probiotic-fed group. Ten minutes post-respiratory challenge, the percent change in pO2 levels (*p* = 0.076, Figure 6) also tended to be less in the probiotic group. However, there were no differences between groups in these lung function assessments by repeated measures analysis.

Taken together, these lung function data suggest that probiotic-fed pigs developed less severe reactions to the respiratory challenge and, when negatively affected, returned faster to pre-challenge status. By the end of the 30 minute monitoring period, the majority of lung function assessments for pigs in the probiotic-supplemented group were not different from those recorded as baseline while control animals failed to recover within this period. Also, while not determined to be statistically significant based on repeated measures analyses, there were differences or trends between control and probiotic-fed groups in some lung response measurements (Pp, pO_2_, and pCO_2_) taken 5 and 10 minutes (pO_2_) post-respiratory challenge. Since the greatest response to the respiratory challenge is at these two time-points, there may be physiological relevance to differences or trends at these individual time-points.

Consistent with lung function tests, the average weight of the control pigs' lungs (340.70±19.68 g) was greater than that of the probiotic group, (294.57±6.27 g, *p* = 0.039), suggesting greater cellular infiltrate and/or edema/mucous production in response to allergen. Since there were no differences between groups in numbers or percentages of leukocytes in BAL samples collected 24 hours post-respiratory challenge (data not shown), inflammatory cell infiltration of the lung parenchyma as well as accumulation of fluid may account for the difference noticed in lung weights.

### Probiotic supplementation modulated the cytokine responses of ASA-stimulated PBMCs from ASA-sensitized pigs

**Figure 7:**
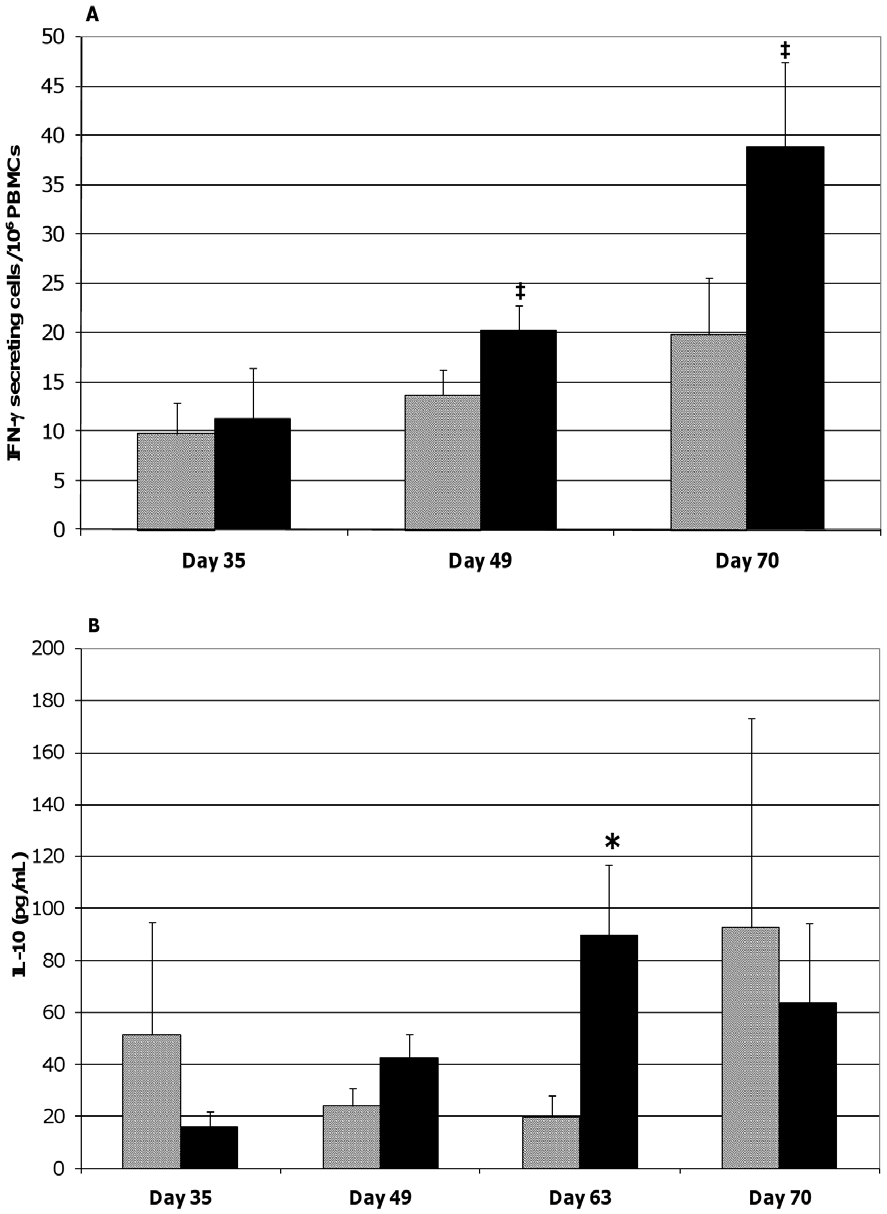
Cytokine production by ASA-stimulated PBMCs. PBMCs were isolated from the blood of ASA-sensitized pigs at the indicated times and stimulated with ASA. The frequencies of ASA-specific IFN-γ secreting PBMCs (A) and the quantities of IL-10 secreted by the PBMCs (B) were determined using an ELISPOT assay and ELISA, respectively. Results are expressed as mean ± SEM for the probiotic-fed (black bar) and control (gray bar) groups of pigs (n = 6 for both groups except at day 70 where n = 5 for the control group). Differences between the average frequency of ASA-specific IFN-γ secreting PBMCs and between the average quantity of IL-10 released by the ASA-stimulated PBMCs of the probiotic-fed and control groups are indicated by ‡ (0.051≤*p* ≤0.1) or * (*p*≤0.05) (Student's t test, unpaired).

Based on ELISPOT assays, the frequency of cells circulating in the peripheral blood of the probiotic-fed pigs and responding to ASA stimulation with IFN-γ secretion tended to temporally increase when measured on study day 49 (*p* = 0.060) and increased on study day 70 (*p* = 0.049) as compared to study day 35 (Figure 7A). Due to technical error, data from study day 63 is not available. A similar pattern in IL-10 production by ASA-stimulated PBMCs from this pig group on study days 49 (*p* = 0.034) and 63 (*p* = 0.059) was noticed (Figure 7B). In contrast, ASA exposure of PBMCs originating from control animals did not result in a significant increase in the frequency of IFN-γ producing cells or in IL-10 production at the respective time-points. Comparisons between groups at individual time-points show that the frequency of ASA-specific IFN-γ producing cells tended to be greater in probiotic-fed pigs compared to control on study days 49 and 70 (*p* = 0.094 and 0.093, respectively, Figure 7A). A similar analysis also demonstrated heightened IL-10 responses by ASA-stimulated PBMCs from probiotic-fed pigs compared to those from the control animals on study day 63 (*p* = 0.033, Figure 7B). Measurements of IL-4 synthesis by ASA-stimulated PBMCs were below the level of quantitation at all time-points. There were no differences by repeated measures analysis in IFN-γ producing cells or in IL-10 levels.

### Pig weights

Pigs were weighed at the study start, study end, and at various time-points throughout the study as an indication of general pig health. Probiotic supplementation did not impact weight gain; pigs in both groups gained weight in a similar fashion (data not shown).

## Discussion

The objective of this study was to determine if the probiotic *Lactobacillus rhamnosus* HN001 could modulate *Ascaris suum-*induced allergic lung response in a pig model. For sensitization of the pigs to ASA, we modified the model described by Fornhem *et al.* [9] where approximately 88% of the pigs receiving three subcutaneous injections of ASA had become sensitized to this allergen. Because our goal was to assess probiotic-induced prevention of allergic lung disease or reduction of initial sensitization, it was necessary to increase the incidence of allergic skin and lung responses to assure 100% of the study population, with no intervention, would become sensitized to the allergen. To this end, the protocol was modified to incorporate, prior to challenge, an extra skin sensitization and two placements of allergen deep in the pigs' lungs via an endotracheal tube. Using this robust method of sensitization, all pigs in the control group developed severe allergic skin reactions and moderate to severe respiratory responses, similar to asthma, upon dermal or aerosol allergen challenge, respectively. Moreover, these alterations in the frequency and location of ASA administration enabled a nearly five-fold reduction in the amount of allergen administered per pig for the respiratory challenge, from the 14 mg utilized by Fornhem *et al* [9] to the 2.5 mg used here. Another departure from the model was the omission of metyrapone prior to and during respiratory challenge with ASA. This drug was used by Fornhem *et al*. to combat stress-induced increases in cortisol levels that inhibit late phase lung responses (9). However, since our interest lies specifically with early phase lung responses and our initial work with metyrapone resulted in pig emesis, metyrapone was not included in our studies.

The respiratory responses of the control pigs after intratracheal challenge with ASA in this study were consistent with those reported by Fornhem *et al* [9]. In both cases, immediate spikes in airway resistance (Figure 4) were accompanied by downward spikes in arterial blood pO_2_ levels (Figure 6) which remained altered compared to baseline through our 30 minute monitoring period [9]. In addition, pleural pressures (not reported by Fornhem *et al*.) in the control group demonstrated the same pattern as was seen in airway resistance (Figure 5).

In contrast, pigs in the probiotic-fed group had less severe lung reactions and recovered more quickly from their adverse responses to the respiratory challenge. It should be noted that the two groups were composed of out-bred pigs that exhibit inherently greater biological variation than would be expected from inbred animals. Yet, we were still able to demonstrate differences or tendencies in lung responses in these small numbers of animals that had undergone an intense allergy induction protocol. While probiotic supplementation decreased the severity of skin responses to challenge with the maximal doses of ASA, dermal reactions to the lowest doses were not impacted. Thus, HN001 supplementation did not prevent sensitization to ASA at the levels that were tested.

It is generally accepted that mechanisms of allergic diseases are characterized by an imbalance in Th1/Th2 immunity favoring Th2 responses coupled with aberrations in immune regulation [16]. Deficient IFN-γ (Th1) responses have been reported in human infants with atopic dermatitis [17] and are also reported to be early markers of atopic dermatitis [18]. Although the role of IFN-γ in allergic lung disease is controversial, reports indicate that low levels of IFN-γ during the first year of life are associated with recurrent wheeze and asthma in later years [19], [20], [21] while the later phases of chronic inflammation are associated with increased levels of IFN-γ [22].

In this swine model of allergy, the allergic response was induced by immunizing pigs with an extract from the roundworm *Ascaris suum* containing allergens known to elicit strong Th2 responses [23] that had been mixed with alum, an adjuvant classically used to further enhance the intensity of Th2 responses. Although the frequency of ASA-specific IFN-γ secreting cells of the control animals remained relatively constant during the last 35 days of the study, pigs in the probiotic-fed group tended to increase the number of these Th1 type responders in their blood. This trend towards a probiotic-associated boost in IFN-γ production in pigs is consistent with results from human infant studies demonstrating probiotic-associated increases in IFN-γ from PBMCs [6], [24], [25] or cord plasma [26] coupled with decreases in allergic outcomes of atopic dermatitis [2], [6], [7] and cow milk allergy [24]. Moreover, in those studies where probiotic supplementation was not efficacious against allergy [3], [4], [5], no probiotic-associated increases in IFN-γ were observed [3], [18], [27]. Together, these studies suggest that a probiotic-promoted increase in IFN-γ production may be one mechanism responsible for the reduced severity of allergic disease in humans. However, to date, decreased asthma development in humans as a result of probiotic-associated increases in IFN-γ production has yet to be demonstrated.

Towards the end of the study, ASA-stimulated PBMCs from pigs in the probiotic-fed group secreted more IL-10 (regulatory cytokine) compared to those of the control group. As with IFN-γ, deficient IL-10 responses are early markers of atopic dermatitis [18] and are reported in infants with this disease [17]. However, there are no reported differences in IL-10 mRNA expression in cells from high risk wheezy infants compared to low risk and healthy infants [21]. Nevertheless, the extent of IL-10 synthesis may be indicative of the type of immune response associated with effective asthma treatment. Indeed, successful allergen immunotherapy and corticosteroid therapy is associated with increased IL-10 production and numbers of Foxp3^+^ T regulatory cells [28]. However, the role of probiotic modulation of IL-10 expression in humans remains unclear; in the aforementioned human probiotic studies, probiotic supplementation either had no effect on IL-10 production or IL-10 production was not determined [3], [25], [26], [27].

This is not the first report of probiotic modulation of allergic lung disease in an animal model. For instance, several mouse models studying *Lactobacillus* species of probiotics have demonstrated dampening of allergic lung responses [29], [30], [31], [32]. However, to our knowledge this is the first report of modulation of allergic lung disease severity by the probiotic *Lactobacillus rhamnosus* HN001 and the first report of probiotic modulation of allergic lung disease severity in a pig model.

In conclusion, using an intense allergy induction protocol in pigs, we demonstrated that *Lactobacillus rhamnosus* HN001 supplementation reduced the severity of allergic lung outcomes and was accompanied by a tendency of increased Th1 (IFN-γ) responses balanced with increased regulatory responses (IL-10 production) to the allergen. While no animal allergy model is completely representative of human allergy, probiotic modulation of immune responses in this pig allergy model demonstrated similar probiotic modulation of immune responses as was seen in human clinical trials, and may predict probiotic modulation of allergic lung responses in humans.
